# A case in which the ileocolic vein draining into the gastrocolic trunk of Henle could be diagnosed preoperatively: a rare anatomical case report

**DOI:** 10.1186/s40792-022-01462-1

**Published:** 2022-06-06

**Authors:** Rie Mizumoto, Mitsuyoshi Tei, Soichiro Mori, Kentaro Nishida, Akinobu Yasuyama, Masatoshi Nomura, Yukihiro Yoshikawa, Toshinori Sueda, Tae Matsumura, Chikato Koga, Hiromichi Miyagaki, Masanori Tsujie, Yusuke Akamaru

**Affiliations:** Department of Surgery, Osaka Rosai Hospital, 1179-3 Nagasonechō, Kita-ku, Sakai, Ōsaka-fu 591-8025 Japan

**Keywords:** Ileocolic vein, Gastrocolic trunk of Henle, Laparoscopic transverse colectomy

## Abstract

**Background:**

Numerous variations in vascular anatomy have been reported in the right colon. The ileocolic vein (ICV) generally drains directly into the superior mesenteric vein (SMV), and is an important landmark for laparoscopic surgery in right colon cancer. We present here a patient with a vascular anomaly of the ICV that was diagnosed on preoperative imaging.

**Case presentation:**

A 65-year-old woman was diagnosed with transverse colon cancer by colonoscopy. Preoperative computed tomography scan showed that the ICV drained into the gastrocolic trunk of Henle (GCT) rather than the SMV. Single-incision laparoscopic transverse colectomy with D3 lymph node dissection was performed, dividing the middle colic vein (MCV) and preserving the right gastroepiploic vein (RGEV), anterior superior pancreaticoduodenal vein (ASPDV), GCT and ICV. The intraoperatively identified venous anatomy was consistent with the preoperative evaluation, and the RGEV, ASPDV and ICV were found to form the GCT.

**Conclusion:**

We report a rare vascular anatomical anomaly that was diagnosed preoperatively, facilitating safe and successful single-incision laparoscopic surgery with D3 lymph node dissection.

## Background

The vascular anatomy of the right colon is reportedly varied. A clear understanding of the vascular anatomy is crucial to ensure surgical safety and to enable sufficient lymph node dissection. Generally, the ileocolic vein (ICV) is present in almost every person [1], and typically directly drains into the superior mesenteric vein (SMV). The gastrocolic trunk of Henle (GCT), which runs along the anterior aspect of the pancreatic head, is considered a common trunk of the colic vein and right gastroepiploic vein (RGEV) and/or the anterior superior pancreaticoduodenal vein (ASPDV). The anatomical venous variation of the ICV draining into the GCT is extremely rare.

Here, we report a rare vascular anatomical anomaly of the ICV draining directly into the GCT that was diagnosed preoperatively on imaging studies. Using this information, we safely performed single-incision laparoscopic transverse colectomy with D3 lymph node dissection. The present case study highlights the importance of preoperative imaging evaluation.

## Case presentation

**Fig. 1:**
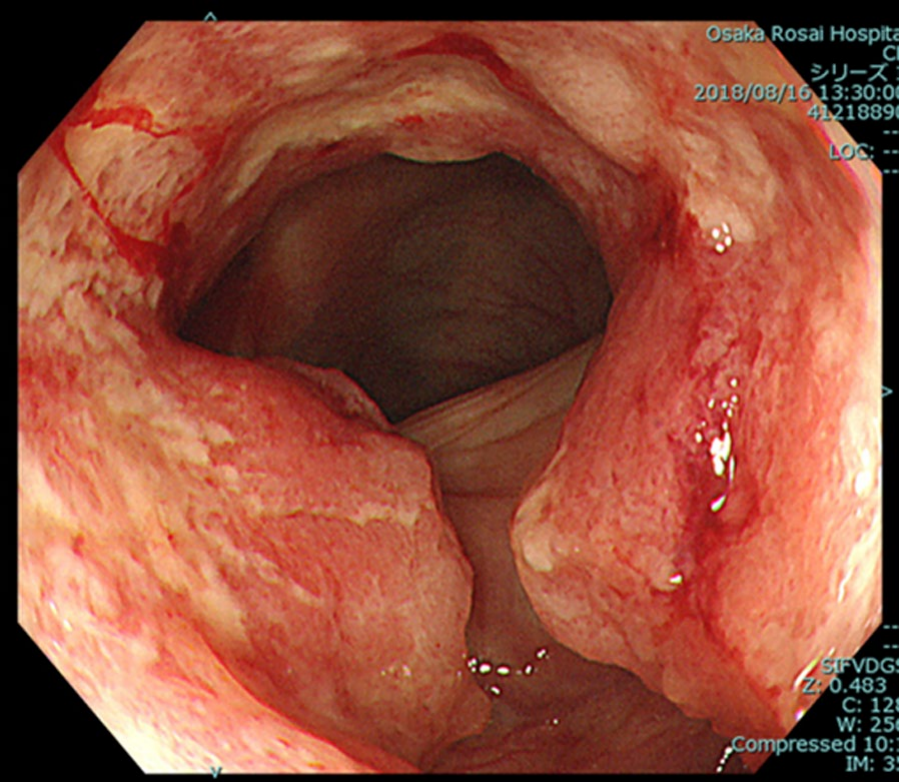
Colonoscopy showing a type III tumor of the transverse colon

**Fig. 2:**
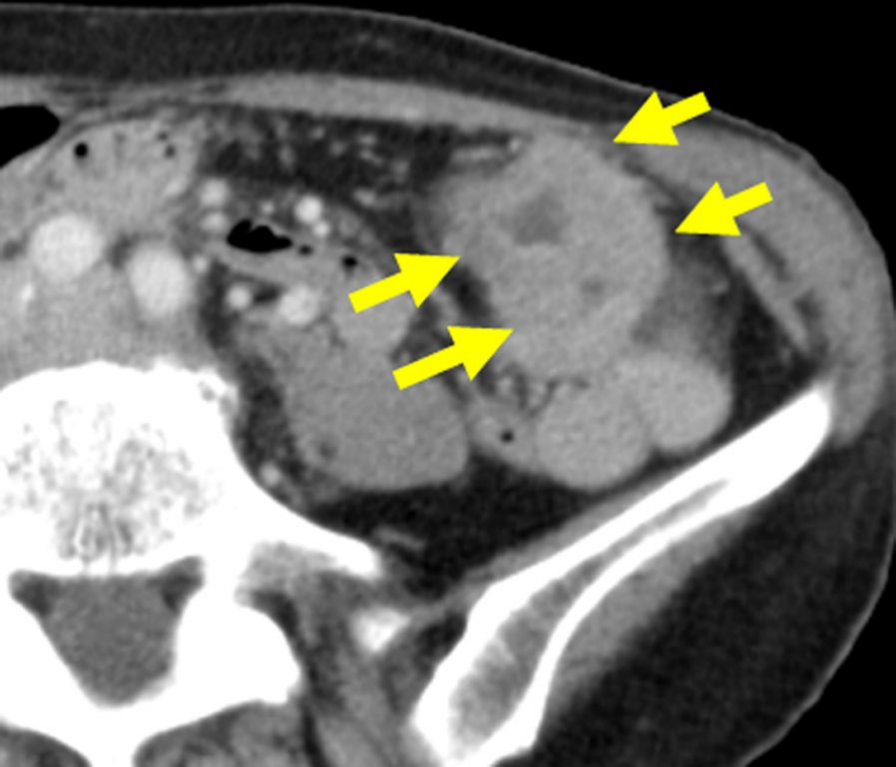
Contrast-enhanced abdominal computed tomographic scan showing sub-circular wall thickening of the transverse colon near the splenic flexure (yellow arrows)

**Fig. 3:**
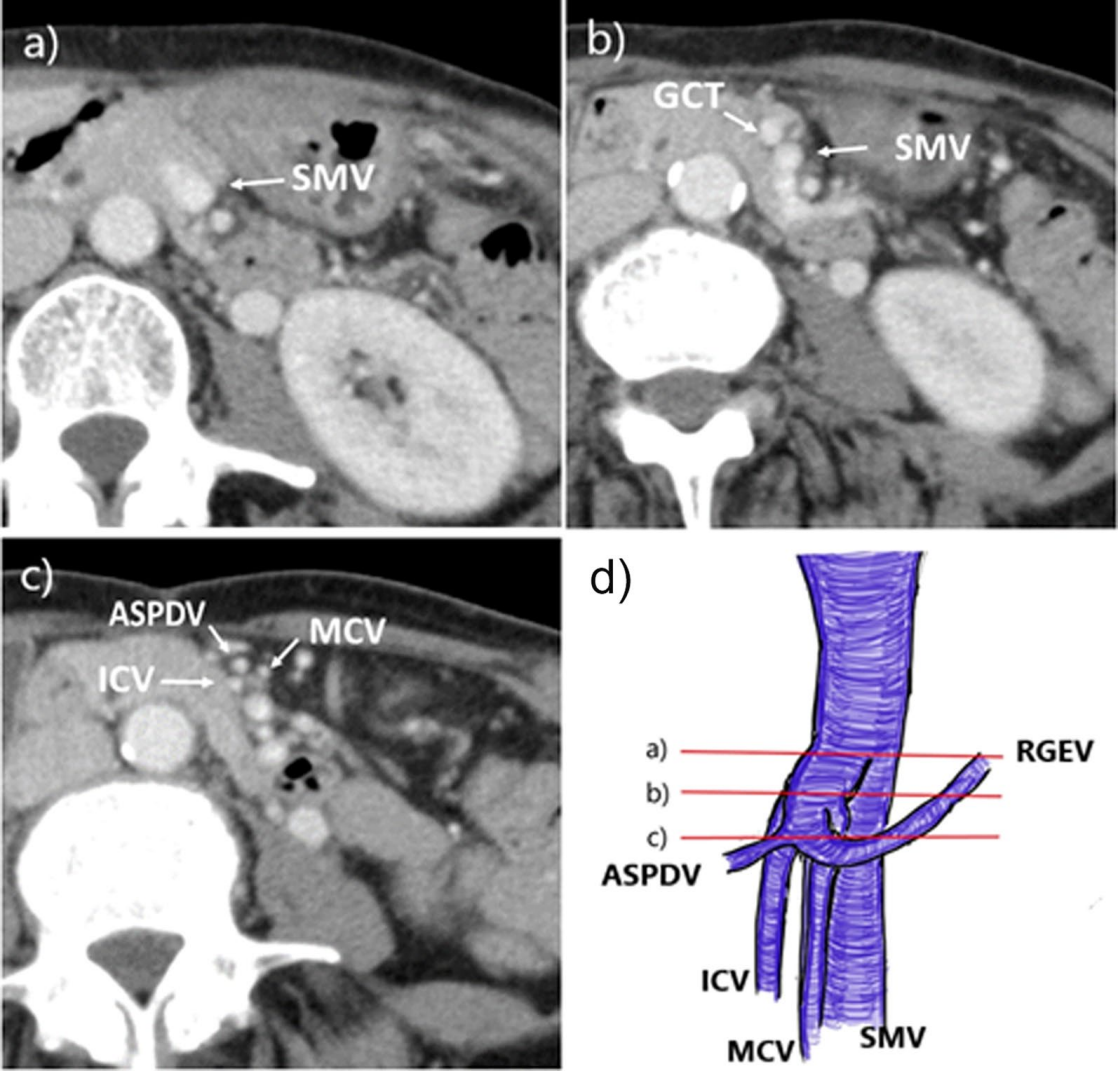
Contrast-enhanced abdominal computed tomographic scan at different levels showing the ileocolic vein (ICV) draining into the gastrocolic trunk (GCT), along with the anterior superior pancreaticoduodenal vein (ASPDV) and right gastroepiploic vein (RGEV). **a** Axial view at the level of line (a). **b** Axial view at the level of line (b). **c** Axial view at the level of line (c). **d** A schema of the portal system. Red line: CT slice level

A 65-year-old woman was being followed up after pulmonary sarcoma surgery. She was admitted to our hospital with the symptom of dyspepsia and because positron emission tomography–computed tomography (PET–CT) showed abnormal accumulation in the transverse colon. Colonoscopy revealed a type II tumor at the left side of the transverse colon (Fig. 1), for which biopsy was performed. Examination of biopsy specimens revealed a well-differentiated tubular adenocarcinoma. The chest and abdomen CT images were acquired with a 64-detector row CT scanner. The CT scan was started at 70 s after the injection of non-ionic contrast agent, and reconstructed thin-slice images that were 0.625 mm thick. The CT revealed sub-circular wall thickening of the transverse colon near the splenic flexure (Fig. 2). Although CT showed no lymph node enlargement and no signs of metastasis, a hypovascular lesion measuring 17 mm was detected in segment 4 of the liver. Additionally, the ICV was seen to drain into the GCT along with the ASPDV and RGEV (Fig. 3), and the ICV pedicle was located cephalad to the pedicle of the middle colic artery (MCA). On laboratory evaluation, the patient’s carcinoembryonic antigen and carbohydrate antigen 19-9 levels were not elevated. Based on these results, she was clinically diagnosed with transverse colon cancer T4a N0 M1a Stage IV according to the TNM classification, for which she underwent single-incision laparoscopic transverse colectomy with D3 lymph node dissection. The operation time was 195 min with minimal blood loss. The final pathological diagnosis was T4a(SE) N1M1a Stage IV.

**Fig. 4:**
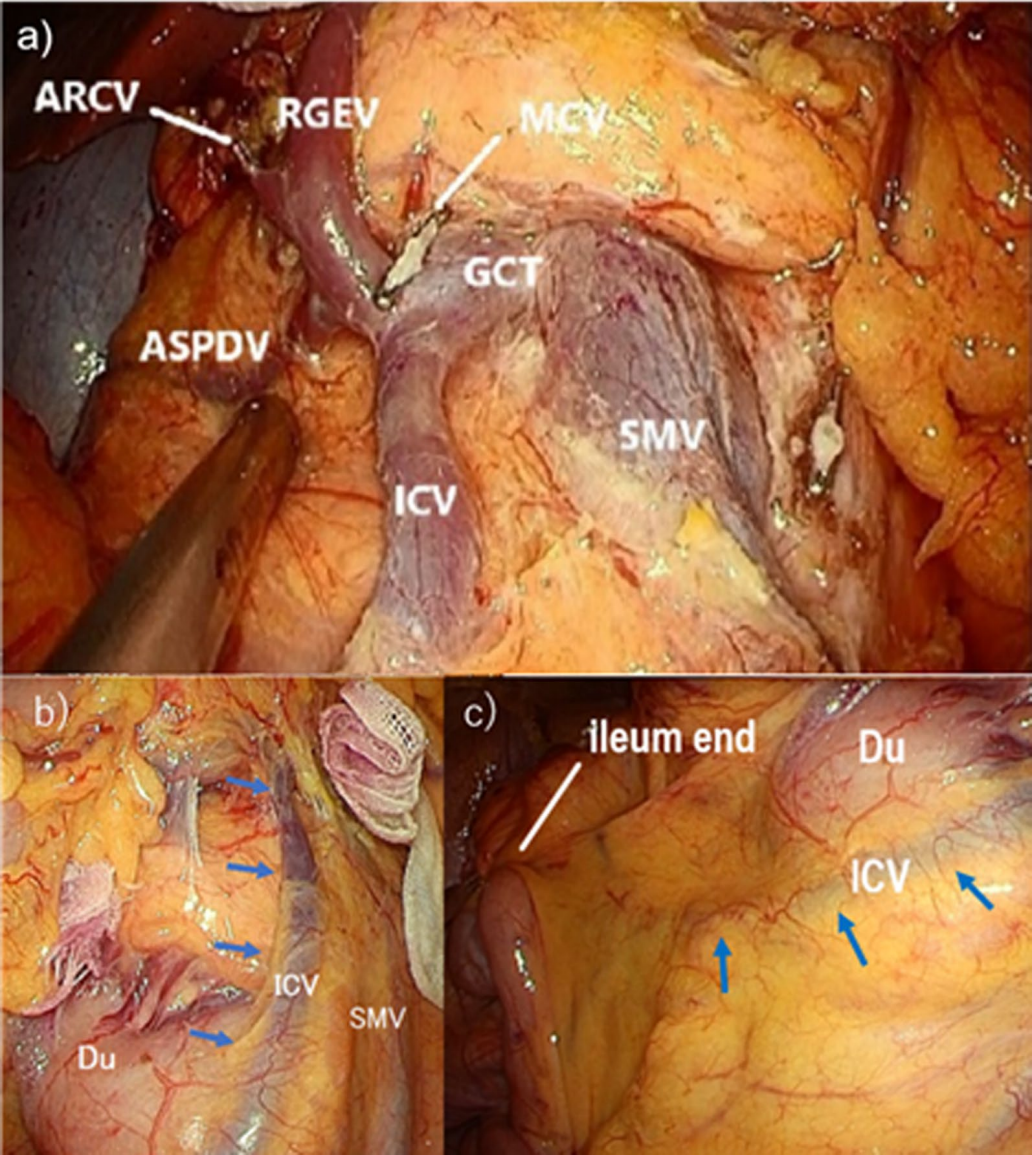
Intraoperative findings. **a** The ileocolic vein (ICV) ran on the ventral side of the duodenum (blue arrow). **b** The ICV ran toward to the GCT on the ventral side of the duodenum and pancreatic head (blue arrow). **c** Situs after lymph node dissection. The ICV drained into the gastrocolic trunk (GCT) with the anterior superior pancreaticoduodenal vein (ASPDV), middle colic vein (MCV) and right gastroepiploic vein (RGEV)

The operation was performed using a cranial-to-caudal (C–C) approach and flip-flap method [2]. The ICV ran towards the GCT on the ventral side of the duodenum and pancreatic head (Fig. 4a–c), which confluence was observed to be cranial to the root of the MCA. The hepatic flexure was mobilized from the second part of the duodenum. Continuing caudally, the dorsal aspect of the ascending mesentery and the mesenteric root were separated from the third part of the duodenum. The greater omentum was separated from the transverse colon, the omental bursa was opened, and the inferior border of the pancreas was exposed. Separating the mesentery from the right side to the left side with maintenance of a layer preserving the prepancreatic fascia, the accessory right colic vein (ARCV) and MCV were divided at the root with preservation of the ICV and GCT, further continuing the dissection of the mesenteric fat above the SMV. The mesenteric fat above the SMV and the superior mesenteric artery (SMA) was dissected from the caudal-tocranial direction, toward the inferior border of the pancreas, and the MCA was divided from the SMA at its root. The MCV, RGEV, ASPDV and ICV were found to form the GCT, confirming the preoperative assessment (Fig. 4c).

The patient had no postoperative complications and was discharged from the hospital 13 days after surgery. The pathological diagnosis was pT4a N2[#221(3/9), #222(2/4), #223(0/10), total *n*(+,5/23)] M1a Stage IV. The proximal and distal margins of the lesion were free of tumor cells. Postoperatively, chemotherapy and subsequent hepatic resection were performed. As of 3 years after the operation, although tumor recurrence in the residual liver has been observed, there is no local or lymph node recurrence.

## Discussion

The present case highlights the usefulness of preoperative imaging examinations. In this patient, careful preoperative imaging evaluation revealed the rare venous anatomy, and reduced the stress of surgeons and improved operative accuracy.

Laparoscopic transverse colectomy for transverse colon carcinoma is considered difficult because of numerous possible variations in the vascular anatomy. Although SMA/SMV variations are common clinical occurrences, our case represents an extremely rare type of anomaly. Normally, the GCT is formed by the RGEV, ASPDV and RCV, and other colic veins might flow into the GCT. According to a previous study [3], the RCV did not drain into the GCT in 15% of patients. In that study, one right colic vein flowed into the GCT in 51.8% of patients, two right colic veins in 11.6%, and one right colic vein and one middle colic vein drained into the GCT in 13.6% of patients.

The ICV generally drains directly into the SMV, and is a good landmark for right colectomy [1]. In our case, the MCV, RGEV, ASPDV and ICV drained into the GCT. The incidence of this variation of the ICV draining directly into the GCT was reported to range from 1.8 to 2.7% using intraoperative imaging [3], cadaver studies [4] and radiologic imaging [5]. Only one previous report by He et al. described the preoperative diagnosis of this type of vascular anomaly [3].

In laparoscopic right colon cancer surgery, various approaches, such as medial-to-lateral [6], caudal-to cranial [7], and cranial [8] approaches have been reported for complete mesocolic excision (CME) with middle colic vessel ligation. However, regardless of the approach used, since the operative view is only from the cranial or caudal side, surgical procedures are limited to only those that can be conducted in front of the partitioned mesocolon, and partitioning of the mesocolon can result in unexpected intraoperative bleeding or injury to other organs. Therefore, we used the cranial approach and flip-flap method [2]. The flip-flap method is a right colon rotating technique, with repeated inversion and restoration of the mobilized right colon according to the anatomical complexity and vascularity, which has been usually adopted in cases with right-sided colon cancer or transverse colon cancer at our institute. The most useful feature of this technique is the ability to develop the optimal operative field according to the anatomical complexity and vascularity in each individual. Moreover, this method shows that the optimal operative field can be developed without partitioning of the mesocolon, regardless of variations in vascular anatomy. This technique enables division of the right and middle colic vessels at their root, without damaging the lymphoadipose tissue to be dissected. These advantages are associated with reductions in both injuries to other organs and unexpected intraoperative bleeding, especially in cases with rare vascular anatomy.

## Conclusion

We diagnosed a rare vascular anatomical anomaly of the ICV draining directly into the GCT in a patient with transverse colon cancer, which enabled safe performance of single-incision laparoscopic transverse colectomy with D3 lymphadenectomy. It is extremely important to preoperatively understand the relevant vascular anatomy and to perform surgery using suitable approaches in each case.

## Data Availability

The data supporting the findings of this study are available within the article.

## Abbreviations

ICV: Ileocolic vein
SMV: Superior mesenteric vein
GCT: Gastrocolic trunk of Henle
MCV: Middle colic vein
RGEV: Right gastroepiploic vein
ASPDV: Anterior superior pancreaticoduodenal vein
PET–CT: Positron emission tomography–computed tomography
MCA: Middle colic artery
ARCV: Accessory right colic vein
C–C approach: Cranial-to-caudal approach
CME: Complete mesocolic excision
